# Endonuclease enrichment TAPS for cost-effective genome-wide base-resolution DNA methylation detection

**DOI:** 10.1093/nar/gkab291

**Published:** 2021-04-27

**Authors:** Jingfei Cheng, Paulina Siejka-Zielińska, Yibin Liu, Anandhakumar Chandran, Skirmantas Kriaucionis, Chun-Xiao Song

**Affiliations:** Ludwig Institute for Cancer Research, Nuffield Department of Medicine, University of Oxford, Oxford OX3 7FZ, UK; Target Discovery Institute, Nuffield Department of Medicine, University of Oxford, Oxford OX3 7FZ, UK; Ludwig Institute for Cancer Research, Nuffield Department of Medicine, University of Oxford, Oxford OX3 7FZ, UK; Target Discovery Institute, Nuffield Department of Medicine, University of Oxford, Oxford OX3 7FZ, UK; Ludwig Institute for Cancer Research, Nuffield Department of Medicine, University of Oxford, Oxford OX3 7FZ, UK; Target Discovery Institute, Nuffield Department of Medicine, University of Oxford, Oxford OX3 7FZ, UK; Ludwig Institute for Cancer Research, Nuffield Department of Medicine, University of Oxford, Oxford OX3 7FZ, UK; Ludwig Institute for Cancer Research, Nuffield Department of Medicine, University of Oxford, Oxford OX3 7FZ, UK; Ludwig Institute for Cancer Research, Nuffield Department of Medicine, University of Oxford, Oxford OX3 7FZ, UK; Target Discovery Institute, Nuffield Department of Medicine, University of Oxford, Oxford OX3 7FZ, UK

## Abstract

Whole genome base-resolution methylome sequencing allows for the most comprehensive analysis of DNA methylation, however, the considerable sequencing cost often limits its applications. While reduced representation sequencing can be an affordable alternative, over 80% of CpGs in the genome are not covered. Building on our recently developed TET-assisted pyridine borane sequencing (TAPS) method, we here described endonuclease enrichment TAPS (eeTAPS), which utilizes dihydrouracil (DHU)-cleaving endonuclease digestion of TAPS-converted DNA to enrich methylated CpG sites (mCpGs). eeTAPS can accurately detect 87% of mCpGs in the mouse genome with a sequencing depth equivalent to 4× whole genome sequencing. In comparison, reduced representation TAPS (rrTAPS) detected less than 4% of mCpGs with 2.5× sequencing depth. Our results demonstrate eeTAPS to be a new strategy for cost-effective genome-wide methylation analysis at single-CpG resolution that can fill the gap between whole-genome and reduced representation sequencing.

## INTRODUCTION

5-Methylcytosine (5mC) and 5-hydroxymethylcytosine (5hmC) are the most common epigenetic marks in eukaryotic genomes, regulating gene expression and numerous cellular processes (1), and they have been intensively studied in normal development and diseases (1,2). For a long time, the only available method and gold standard for base-resolution 5mC and 5hmC analysis was bisulfite sequencing (BS) (3). This method involves bisulfite treatment of DNA to induce deamination of unmodified cytosine to uracil while leaving methylated cytosine intact. Uracil residues are then amplified as thymine during PCR resulting in a C-to-T transition of unmodified cytosine (4,5). Despite its wide application, bisulfite sequencing has two main drawbacks. First, it involves harsh chemical treatment that leads to degradation of a substantial portion of input DNA (6). Second, bisulfite-treated DNA libraries have low sequence complexity due to C-to-T conversion of unmodified cytosine, which constitutes nearly 95% of all cytosine in the genome. Low complexity leads to lower sequencing quality, decreased mapping rate and an overall increase in sequencing costs (7). Recently, we developed a bisulfite-free and base-resolution DNA methylation sequencing method called TET-assisted pyridine borane sequencing (TAPS) (8–10). In the TAPS method, 5mC and 5hmC are oxidised by ten-eleven translocation (TET) proteins to 5-carboxylcytosine (5caC) and subsequently reduced to dihydrouracil (DHU) by pyridine borane. DHU is then amplified and sequenced as thymine (T) during final sequencing. TAPS sequencing introduces significantly less DNA damage compared to bisulfite sequencing and has improved sequencing results, for example, better sequence quality, mapping rate and coverage (8).

Compared to whole-genome bisulfite sequencing (WGBS), whole-genome TAPS (wgTAPS) reduces the sequencing cost by half (8). However, the cost of whole-genome sequencing is still prohibitive for many projects, especially considering 5mC and 5hmC accounts for only ∼4% of all cytosine residues the mammalian genome (11) and around 65–80% of reads generated by short read whole-genome sequencing do not contain any methylated CpG sites (mCpGs) (12). To reduce sequencing cost, Reduced Representation Bisulfite Sequencing (RRBS) is a widely used method where CpG-rich regions are enriched by restriction endonucleases prior to bisulfite treatment (13). However, it covers only a small proportion of CpG sites in specific sequence contexts and therefore does not yield a comprehensive methylation picture. Therefore, there is a need for approaches which achieve better coverage of mCpGs for lower cost.

The newly developed TAPS method offers opportunities for the development of new enrichment-based strategies. Not only can TAPS work with restriction endonucleases to enrich CpG-rich regions in reduced representation TAPS (rrTAPS), analogous to RRBS, but more importantly, some endonucleases were known to cleave the TAPS-converted product, DHU, in DNA (14,15). We envisioned that such endonuclease enrichment of methylation by direct digestion of TAPS-converted DNA, an approach impossible for bisulfite sequencing, could help to capture genome-wide CpG sites regardless of their sequence context compared to traditional reduced representation approaches. Here, we present endonuclease enrichment TAPS (eeTAPS) as a new strategy that combines TAPS with DHU-cleaving endonuclease digestion. In eeTAPS, DHU sites in TAPS-converted DNA are digested by USER enzyme (Uracil-Specific Excision Reagent, which is a mixture of uracil DNA glycosylase (UDG) and Endonuclease VIII). Cleaved fragments are then converted into a sequencing library in which the beginning and the end of each fragment correspond to the methylation sites. This allows the mCpGs to be enriched genome-wide while the vast majority of the genome with no methylation is depleted.

We applied eeTAPS to model DNA and genomic DNA (gDNA) from mouse embryonic stem cells (mESCs) and compared this method with both wgTAPS and rrTAPS. We showed that eeTAPS is a cost-effective approach to directly detect whole-genome mCpGs and offers an attractive alternative to the coverage-biased reduced representative sequencing and the costly whole-genome sequencing.

## MATERIALS AND METHODS

### Preparation of spike-in controls

A 4 kb spike-in control was prepared by PCR amplification of the pNIC28-Bsa4 plasmid (Addgene, cat. no. 26103) in a reaction containing 1 ng DNA template, 0.5 μM primers and 1X Phusion High-Fidelity PCR Master Mix with HF Buffer (Thermo Scientific). Primer sequences are listed in Supplementary Table S1. The PCR product was purified by Zymo-IC column (Zymo Research) and methylated by HpaII methyltransferase (New England Biolabs) for 2 h at 37°C in a 50 μl reaction. Methylated product was purified with 1X Ampure XP beads (Beckman Coulter) according to the manufacturer's protocol. Fully CpG-methylated λ-DNA was prepared by methylation of unmethylated λ-DNA (Promega) with M.SssI enzyme (New England Biolabs) as described previously (16). Bisulfite sequencing of the 4 kb spike-in was downloaded from PRJNA588716 (9).

### Preparation of carrier DNA

Carrier DNA was prepared by PCR amplification of the pNIC28-Bsa4 plasmid (Addgene, cat. no. 26103) in a reaction containing 1 ng DNA template, 0.5 μM primers and 1X Phusion High-Fidelity PCR Master Mix with HF Buffer (Thermo Scientific). Primer sequences are listed in Supplementary Table S1. The PCR product was purified by Zymo-IC column (Zymo Research), fragmented by Covaris M220 and purified on 0.9× Ampure XP beads to select for 200–500 bp fragments.

### Expression and purification of mTet1CD

The mTet1CD catalytic domain (NM_001253857.2, 4371–6392) with N-terminal Flag-tag was cloned into pcDNA3-Flag between the KpnI and BamH1 restriction sites (8). For protein expression, 1 mg plasmid was transfected into 1 l of Expi293F cell culture at density 1 × 10^6^ cells ml^−1^ and cells were grown for 48 h at 37 °C, 170 r.p.m. and 5% CO_2_. Subsequently, cells were harvested by centrifugation, re-suspended in the lysis buffer containing 50 mM Tris–Cl pH 7.5, 500 mM NaCl, 1× cOmplete Protease Inhibitor Cocktail, 1 mM PMSF, 1% Triton X-100 and incubated on ice for 20 min. Cell lysate was then clarified by centrifugation for 30 min at 30 000*g* and 4°C. Collected supernatant was purified on ANTI-FLAG M2 Affinity Gel and pure protein was eluted with buffer containing 20 mM HEPES pH 8.0, 150 mM NaCl, 0.1 mg ml^−1^ 3× Flag peptide, 1× cOmplete Protease Inhibitor Cocktail, 1 mM PMSF. Collected fractions were concentrated and buffer exchanged to the final buffer containing 20 mM HEPES pH 8.0, 150 mM NaCl and 1 mM dithiothreitol. Concentrated protein was mixed with glycerol (30% v/v), frozen in liquid nitrogen and aliquots were stored at −80°C.

### mESCs culture and isolation of genomic DNA

E14 mESC were cultured on gelatin-coated plates in DMEM (Invitrogen) supplemented with 15% FBS (Gibco), 2 mM l-glutamine (Gibco), 1% non-essential amino acids (Gibco), 1% penicillin/streptavidin (Gibco), 0.1 mM β-mercaptoethanol (Sigma), 1000 units ml^−1^ leukaemia inhibitory factor (Millipore), 1 μM PD0325901 (Stemgent) and 3 μM CHIR99021 (Stemgent). Cultures were maintained at 37°C and 5% CO_2_ and passaged every 2 days.

For isolation of genomic DNA, cells were harvested by centrifugation for 5 min at 1000*g* and room temperature. DNA was extracted with Quick-DNA Plus kit (Zymo Research) according to the manufacturer's protocol.

### mTet1CD oxidation

Genomic DNA (up to 200 ng) was incubated in a 50 μl reaction containing 50 mM HEPES buffer (pH 8.0), 100 μM ammonium iron(II) sulfate, 1 mM α-ketoglutarate, 2 mM ascorbic acid, 1 mM dithiothreitol, 100 mM NaCl, 1.2 mM ATP and 4 μM mTet1CD for 80 min at 37 °C. After that, 0.8 U of Proteinase K (New England Biolabs) were added to the reaction mixture and incubated for 1 h at 50°C. The product was cleaned up on Bio-Spin P-30 Gel Column (Bio-Rad) and 1.8× AMPure XP beads following the manufacturer's instruction.

### eeTAPS

mESC genomic DNA (200, 50, 10 or 1 ng) was spiked with 0.05% 4kb control methylated in CCGG sequence context and oxidised by mTet1CD as described above. Subsequently, oxidized DNA samples in 35 μl of water were reduced in a 50 μl reaction containing 600 mM sodium acetate solution (pH 4.3) and 1 M pyridine borane for 16 h at 37 °C and 850 r.p.m. in an Eppendorf ThermoMixer. The product was purified using Zymo-Spin columns. Converted samples were digested in a 20 μl reaction containing 2 U of USER enzyme (New England Biolabs) in CutSmart buffer for 1 h at 37°C and size-selected on 0.35×–1× Ampure XP beads. End-repair and A-tailing reactions, and ligation of Illumina Multiplexing adapters were prepared with KAPA Hyper kit according to the manufacturer's protocol. To prepare the control library, 200 ng of unconverted mESC gDNA with spike-in controls was digested by USER enzyme, size-selected and used for library construction as described above. The final sequencing libraries were amplified with KAPA HiFi HotStart ReadyMix for 6 cycles (for 200 ng input), 8 cycles (50 ng input), 10 cycles (10 ng input) or 14 cycles (1 ng input) and size-selected on 0.35×-1× Ampure XP beads. Final libraries were paired-end 80 bp sequenced on a NextSeq 500 sequencer (Illumina) together with other sequencing libraries.

### rrTAPS

1 μg mESC gDNA was spiked with 1% CpG-methylated λ-DNA and digested by Fast digest Msp1 enzyme (Thermo Scientific) in 50 μl reaction for 30 min at 37°C. Digested DNA was purified by the phenol/chloroform precipitation method. End-repair and A-tailing reactions, and ligation of Illumina Multiplexing adapters were prepared with NEBNext^®^ Ultra™ II DNA Library Prep Kit according to the manufacturer's protocol. The ligated library was then purified on 1.6× Ampure XP beads and run on a 1% agarose gel. DNA fragments from 100 to 400 bp were excised and purified by Monarch^®^ DNA Gel Extraction Kit following the manufacturer's protocol. The adapter-ligated sample was spiked with 100 ng of carrier DNA and double oxidised by mTet1CD as described above. Oxidized DNA in 35 μl of water was reduced in a 50 μl reaction containing 600 mM sodium acetate solution (pH 4.3) and 1 M pyridine borane for 16 h at 37 °C and 850 r.p.m. in an Eppendorf ThermoMixer. The product was purified using Zymo-Spin columns. The final sequencing library was amplified with KAPA HiFi Uracil (+) Master Mix for 6 cycles and purified on 1× Ampure XP beads. Final libraries were paired-end 80 bp sequenced on a NextSeq 500 sequencer (Illumina) together with other sequencing libraries.

### Screening for DHU digesting endonucleases

1 μg mESC gDNA was enzymatically oxidised by mTet1CD as described above. Subsequently, oxidized DNA in 35 μl of water was reduced in a 50 μl reaction containing 600 mM sodium acetate solution (pH 4.3) and 1 M pyridine borane for 16 h at 37 °C and 850 r.p.m. in an Eppendorf ThermoMixer. The product was purified using Zymo-Spin columns. 40 ng of TAPS converted or unconverted DNA were then digested by the following enzymes according to the manufacturers’ protocols (all from New England Biolabs): USER (Cat. No. M5505S), Endonuclease IV (Cat. No. M0304S), Tma Endonuclease III (Cat. No. M0291S), Endonuclease V (Cat. No. M0305S), UDG (Cat. No. M0280S), Tth Endonuclease IV (Cat. No. M0294S), Fpg (Cat. No. M0240S), Endonuclease III (Nth) (Cat. No. M0268S), Endonuclease VIII (Cat. No. M0299S), APE1 (Cat. No. M0282S). Digestion products were purified on 1.8× Ampure XP beads following the manufacturer's instructions and 10 ng of each product were run on a 2% agarose gel.

### Preparation of model DNA for DHU cleavage efficiency quantification

Short model DNA with single mCpG in different sequence contexts (ACG, CCG, GCG and TCG) were produced by the annealing and extension method. Synthetic oligos used for preparation of model DNA were purchased from IDT and detailed sequences are listed in Supplementary Table S2. Briefly, 10 μM of top and bottom strand oligos were annealed in the annealing buffer containing 10 mM Tris–Cl (pH 8.0), 50 mM NaCl and 0.1 mM EDTA (pH 8.0) with the following program: 2 min at 95°C, 140 cycles of 20 s at 95°C (decrease temperature 0.5°C every cycle) and hold at 4°C. Extension was performed in the NEB buffer 2 with 0.4 mM dNTPs (dATP/dGTP/dTTP/dCTP) and 5 U of Klenow Polymerase (NEB) for 1 h at 37°C. After the reaction, the spike-in control was purified on Zymo-Spin IC column (Zymo Research).

### DHU cleavage efficiency quantification

200 ng of model DNA (TCG context) was oxidised by mTet1CD and reduced by pyridine borane as described above. 40 ng of TAPS converted or unconverted model DNA (negative control) was then digested by the following enzymes according to the manufacturers’ protocols (all from New England Biolabs): USER (Cat. No. M5505S), Tma Endonuclease III (Cat. No. M0291S), Fpg (Cat. No. M0240S), Endonuclease III (Nth) (Cat. No. M0268S). Digestion products were purified on Zymo-Spin IC column (Zymo Research) with Oligo Binding buffer (Zymo Research) following the manufacturer's instructions and 5 ng of each product was run on a 2% agarose gel. Cleavage efficiency was estimated based on the gel band intensity corresponding to cleaved and un-cleaved DNA substrate.

To compare DHU cleavage efficiency in different sequence contexts 200 ng of model DNA with 5mC in ACG, CCG, GCG or TCG sequence context was oxidised by mTet1CD and reduced by pyridine borane as described above. 40 ng of TAPS converted or unconverted model DNA (negative control) was then digested by USER (Cat. No. M5505S) according to manufacturer's protocol. Digestion products were purified on Zymo-Spin IC column (Zymo Research) with Oligo Binding buffer (Zymo Research) following the manufacturer's instructions and 5 ng of each product was run on a 2% agarose gel. Cleavage efficiency was estimated based on the gel band intensity corresponding to cleaved and un-cleaved DNA substrate.

### Data analysis for eeTAPS

Raw sequenced reads were processed with TrimGalore (https://www.bioinformatics.babraham.ac.uk/projects/trim_galore/) to perform adapter and quality trimming with the following parameters: –paired –length 35. Cleaned reads were aligned using bwa mem 0.7.17-r1188 (17) with default parameters. For the 4 kb model DNA, the pNIC28-Bsa4 sequence from 2627 to 6911 was used as reference. For mESC gDNA, the mm9 genome was used as reference. Only properly mapped read pairs (Read 1 with flag assigned as 83 or 99) were extracted to compute coverage with bedtools v2.27.1 (18) for both endpoints and read-through of the whole fragments, and un-cleaved sites were also taken into consideration when calculating the cleavage fraction. The detailed computational pipeline to analyze eeTAPS can be found here https://gitlab.com/jfeicheng/userenrich. Two technique replicates were sequenced with eeTAPS, which were sequenced at depth 6× and 4×, respectively. When analyzing the effect of sequence depth on eeTAPS, the alignment files from two replicates were merged and then sub-sampled by fraction from 0.1 to 1 with samtools view (19).

### Data analysis for rrTAPS

Raw sequenced reads were processed with seqtk (https://github.com/lh3/seqtk) trimfq -b 2 to trim 2 bp from the left of each read. Astair 3.2.7 was used to process rrTAPS (8). Cleaned reads were aligned using astair align with mm9 genome as reference. mCpGs were extracted with astair call.

### Comparison **of wgTAPS, eeTAPS and rrTAPS** in mESC

wgTAPS data was downloaded from GSE112520 (8). CpG sites covered with at least 5 reads were considered as covered CpG sites in wgTAPS and rrTAPS, and CpG sites with at least 1 read were consider as covered CpGs in eeTAPS. The mCpGs was defined according to the following criteria: CpG methylation level >0.1. The genome was divided into non-overlapping 100 kb windows with bedtools (18). The CpG island track was downloaded from http://hgdownload.soe.ucsc.edu/goldenPath/mm9/database/cpgIslandExt.txt.gz. The gene annotation file was downloaded from http://hgdownload.soe.ucsc.edu/goldenPath/mm9/database/refGene.txt.gz. Average methylation was used to assign methylation in each region for both wgTAPS and eeTAPS. Gene expression data from the e14 mESC cell line was taken from GEO entry GSE72855 (20) and used to categorize genes into four groups according to their expression levels.

## RESULTS

### Development of eeTAPS

In order to enrich mCpGs for sequencing following the TAPS reaction, we needed to identify an endonuclease that would specifically cleave DHU, the product of TAPS-converted methylated cytosine. We tested 10 commercially available endonucleases with known ability to digest DHU (14,15) or structurally similar nucleotides (uracil, 5-hydroxymethyluracil, dihydrothymine) and found that some of them, including USER, Endonuclease VIII, Endonuclease III and Fpg, can indeed cleave TAPS-converted DNA at various efficiencies, while others such as APE 1 and UDG cannot cleave TAPS-converted DNA (Supplementary Figure S1A). We then quantified the cleavage efficiencies of these enzymes using model DNA with single mCpG following TAPS conversion. None of enzymes exhibited unspecific digestion of unconverted DNA and USER showed the highest cleavage efficiency of the converted DNA (85.0%, Supplementary Figure S1B), which was chosen for further method development. Furthermore, we evaluated the USER digestion efficiency in different sequence context and showed that it exhibited high efficiencies across all NCG sequence context (range from 82.6% in TCG sites to 92.4% in ACG sites, Supplementary Figure S1C).

**Figure 1.**
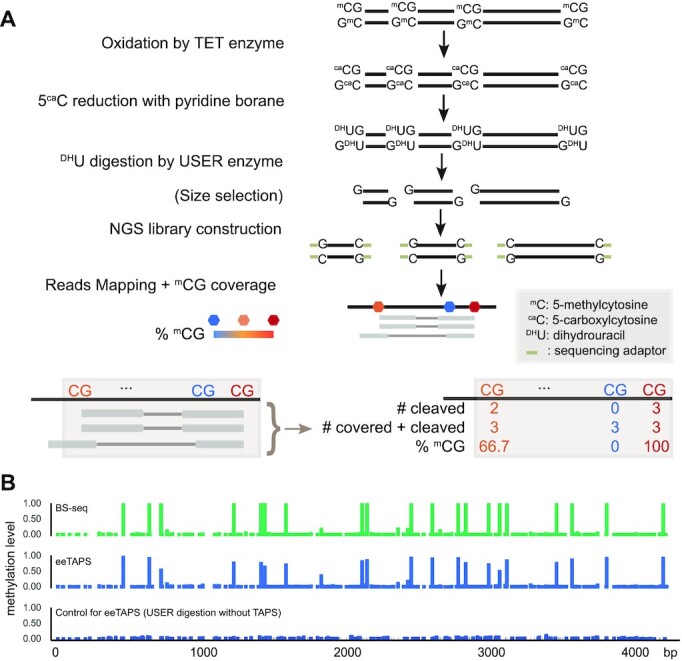
Endonuclease enrichment TAPS (eeTAPS). (**A**) Schematic of eeTAPS (top) and computational measurement of CG methylation level (bottom). 5-methylcytosine (^m^C) was first converted to dihydrouracil (^DH^U) with TAPS and then enriched through USER digestion. Size selected DNA fragments were then amplified by PCR and prepared into sequencing library. Following reads alignment, CG methylation level was then calculated as the number of reads that are cleaved at each CpG site divided by the total number of reads cleaved at or covering each CpG site. (**B**) Validation of eeTAPS on a 4 kb model DNA. The tracks from top to bottom indicate the methylation level measured in bisulfite sequencing (BS-seq), eeTAPS and a control for eeTAPS. In the eeTAPS control, USER enzyme was used to digest DNA without TAPS conversion.

We then combined TAPS conversion with USER digestion to enrich mCpGs. First, we converted un-fragmented genomic DNA (gDNA) from mouse embryonic stem cells (mESCs) with TAPS and digested with USER. Cleavage resulted in DNA fragments ranging from 100 bp to 10 kb (Supplementary Figure S1D). Presumably, the shorter fragments originate from densely methylated regions and the long fragments originate from sparsely methylated parts of the genome. Since Illumina sequencing is biased towards short DNA fragments, as a proof-of-concept to include a broad range of fragments (represents a broad range of methylation density), we size-selected the fragmented DNA to remove very short (< 100 bp) and very long (> 1 kb) fragments and prepared an Illumina sequencing library (Figure 1A, Supplementary Figure S1D). We demonstrated that final eeTAPS library had broad insert size distribution with 46.3% of fragments below 200 bp (Supplementary Figure S1E). To identify and quantify mCpGs, we developed a computational pipeline. The methylation level is calculated as the number of reads that are cleaved at each CpG site divided by the total number of reads cleaved at or covering each CpG site (Figure 1A).

To evaluate the performance of eeTAPS, we prepared a 4 kb spike-in model DNA with all CpGs in CCGG sites methylated by HpaII methytransferase, which also generated some low-level CpG methylation in off-target non-CCGG sites (Supplementary Table S3). We obtained excellent agreement between eeTAPS methylation and bisulfite methylation in the model DNA (Pearson correlation coefficient (*r*) = 0.98) (Supplementary Figure S1F, Figure 1B and Supplementary Table S3). On the other hand, in a control sample where USER enzyme was used to digest non-TAPS converted 4 kb model DNA, none of the CpGs were detected with significant methylation (Figure 1B, Supplementary Table S3). Together, these results demonstrated the high efficiency and specificity of eeTAPS in detecting DNA methylation.

### eeTAPS on mESC

Having demonstrated the ability for eeTAPS on model DNA, we next sought to utilize eeTAPS to profile CpG methylation in mESCs gDNA (Supplementary Table S4). We first sequenced eeTAPS to 6× depth. eeTAPS is proposed to be a cost-efficient methodology since it will enrich mCpGs. Indeed, we found that 84.6% of fragments in eeTAPS end with C/G (Supplementary Figure S2A). Further analysis on the distance between cleaved sites and the nearest CpG identified that 72.7% of cleaved events occurred on CpG (Supplementary Figure S2B).

**Figure 2.**
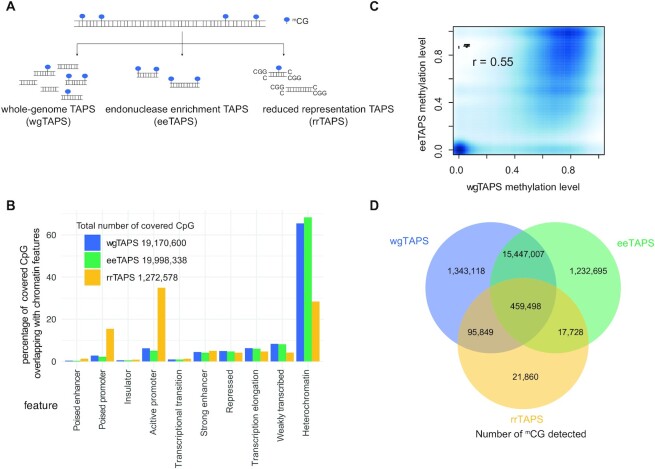
Comparison of wgTAPS, eeTAPS and rrTAPS on mESC DNA. (**A**) Diagram showing the genomic fragmentation method for wgTAPS, eeTAPS and rrTAPS. In wgTAPS, genomic DNA is randomly fragmented, while for eeTAPS and rrTAPS, fragmentation happens specifically at mCpGs (^m^CG) and CCGG sites respectively. (**B**) Barplot showing the percentage of CpG sites covered by wgTAPS, eeTAPS and rrTAPS overlapping with different chromatin features. The chromatin features were defined in previous study (21). (**C**) Smoothed density plot showing the methylation level determined by wgTAPS and eeTAPS at single CpG-resolution. Only CpG sites covered within both wgTAPS and eeTAPS were taken into consideration. Pearson correlation coefficient was shown on the top of the plot. (**D**) Venn plot showing the overlap of detected mCpG sites in wgTAPS, rrTAPS and eeTAPS. CpG sites with methylation level >0.1 were defined as mCpGs.

To further illustrate this point, we compared eeTAPS with wgTAPS and rrTAPS (Figure 2A, Supplementary Table S4). First, we compared the number of CpG sites that are covered in all three methods (covered CpGs were defined as CpG sites with the minimal depth of 5 in wgTAPS and rrTAPS, and CpG sites with the minimal depth of 1 in eeTAPS). Unsurprisingly, wgTAPS and eeTAPS covered the majority of CpG sites (19.2 M and 20.0 M sites respectively; 91.3% and 95.2% of total CpGs respectively), while rrTAPS only covered ∼1.3 M sites (6.1% of total CpGs) (Figure 2B). To further compare the genomic regions covered by these assays, we mapped the covered sites to different genomic regions (21). Intergenic methylation such as those in distal regulatory elements are also known to be important for gene regulation (12). We found that wgTAPS and eeTAPS share a similar broad feature distribution, with the majority of covered CpG sites lying in heterochromatin (65.4% and 68.3%, respectively), while rrTAPS is biased towards promoter regions (34.9% of covered CpGs) (Figure 2B). rrTAPS, as a quantitative method, showed excellent correlation with wgTAPS (r = 0.90, Supplementary Figure S2C) among the covered CpG sites as expected. eeTAPS, as an enrichment method, still showed good correlation with wgTAPS (*r* = 0.55, Figure 2C) on CpG level.

Next, we compared the mCpGs that are covered in different assays. As expected, eeTAPS and wgTAPS show high agreement in terms of the sites that are defined as mCpGs (covered CpG sites with methylation level >0.1 were defined as mCpGs in both eeTAPS and wgTAPS; 91.7% mCpG sites detect by wgTAPS are also detect by eeTAPS, Figure 2D), while rrTAPS only detect about 3.5% of mCpGs (Figure 2D). Furthermore, eeTAPS showed high reproducibility with 87.6% mCpGs observed between replicates (Supplementary Figure S2D). Collectively, these analyses support that eeTAPS can accurately and robustly detect mCpG sites at a whole-genome scale and can be a powerful semi-quantitative tool for measuring methylation at single-CpG resolution.

### Comparison of eeTAPS and wgTAPS on genomic features

**Figure 3.**
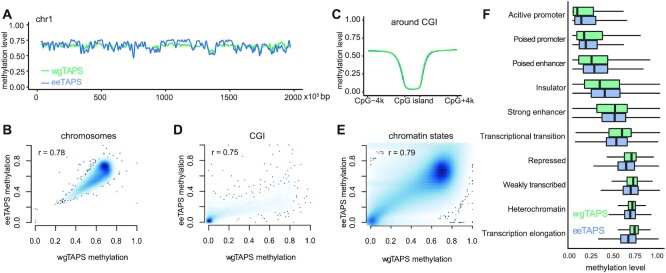
Methylation profiling in different genomic features with eeTAPS. (**A**) Methylation level measured by both eeTAPS (blue) and wgTAPS (green) in chromosome 1 of the mESC. 100 kb windows were used, and a moving average value was calculated using the movAvg2 function in R with bw = 10. (**B**) Density plot showing methylation level correlation between eeTAPS and wgTAPS in chromosomes bins. A 100 kb window was used to calculate the average methylation level in each bin. Pearson correlation coefficient was shown on the top of the plot. (**C**) Average methylation level across CpG Islands (CGI) and the 4 kb flanking regions for eeTAPS (blue) and wgTAPS (green). (**D**) Density plot showing methylation level correlation between eeTAPS and wgTAPS in CpG Islands. Pearson correlation coefficient was shown on the top of the plot. (**E**) Density plot showing methylation level correlation between eeTAPS and wgTAPS in different chromatin features. The chromatin states map was previously annotated based on ENCODE ChIP-seq data with ChromHMM (21). For each region, the CpG methylation level was calculated by taking the arithmetic average methylation level across all covered CpG sites. All regions annotated in the chromatin states map were included in the comparison, and Pearson correlation coefficient was computed between the methylation level in wgTAPS and eeTAPS. (**F**) Boxplot showing the distribution of methylation level across all chromatin features as measured by eeTAPS (blue) and wgTAPS (green).

We then compared the methylation pattern across different genomic features between the 6x eeTAPS and 27x wgTAPS. To quantify methylation level in a region, the arithmetic average methylation of all CpG sites within the region was used in both wgTAPS and eeTAPS. As expected, eeTAPS and wgTAPS showed highly correlated chromosome-wide methylation patterns (Figure 3A, B). We further investigated the regions which showed less consistent methylation between eeTAPS and wgTAPS, and we found that eeTAPS tends to overestimate methylation on regions of high mCpGs density and underestimate methylation on regions of low mCpGs density (Supplementary Figure S3A). CpG islands (CGIs) are known to be depleted of DNA methylation, and these are reflected in both eeTAPS and wgTAPS (Figure 3C). Correlation of the methylation level on CGIs measured using eeTAPS and wgTAPS was 0.75, which further indicates that eeTAPS can accurately capture the CpG methylation state in various features (Figure 3D).

Previous studies reveal that DNA methylation in promoter regions is generally anti-correlated with gene expression (22,23). We categorised genes into four groups according to their expression levels and plotted the average methylation from 4 kb upstream of the transcription start site (TSS) to 4 kb downstream. As we expected, using both eeTAPS and wgTAPS we found that highly expressed genes tend to have lower methylation levels, while genes with lower expression levels have higher methylation levels (Supplementary Figure S3B). We also compared the methylation distribution in different chromatin features as defined previously (21). Correlation analysis showed consistent methylation between wgTAPS and eeTAPS across all annotated regions which were defined in chromatin state map (Figure 3E). Consistent with previous research, heterochromatin regions are highly methylated while promoter regions in euchromatin are normally depleted of CG methylation (Figure 3F). The methylation difference between eeTAPS and wgTAPS among the annotated regions is also dependent on the density of mCpGs (Supplementary Figure S3C), which is consistent with the observation on chromosomal comparison. For the regions which are more likely to have low mCpGs density such as active promoters and insulators, eeTAPS tends to overestimate methylation (Supplementary Figure S3D). For regions which are more likely to have high mCpGs density such as transcriptional transition and transcription elongation regions, eeTAPS tends to underestimate methylation (Supplementary Figure S3D).

### Application of eeTAPS on low-input samples

**Figure 4.**
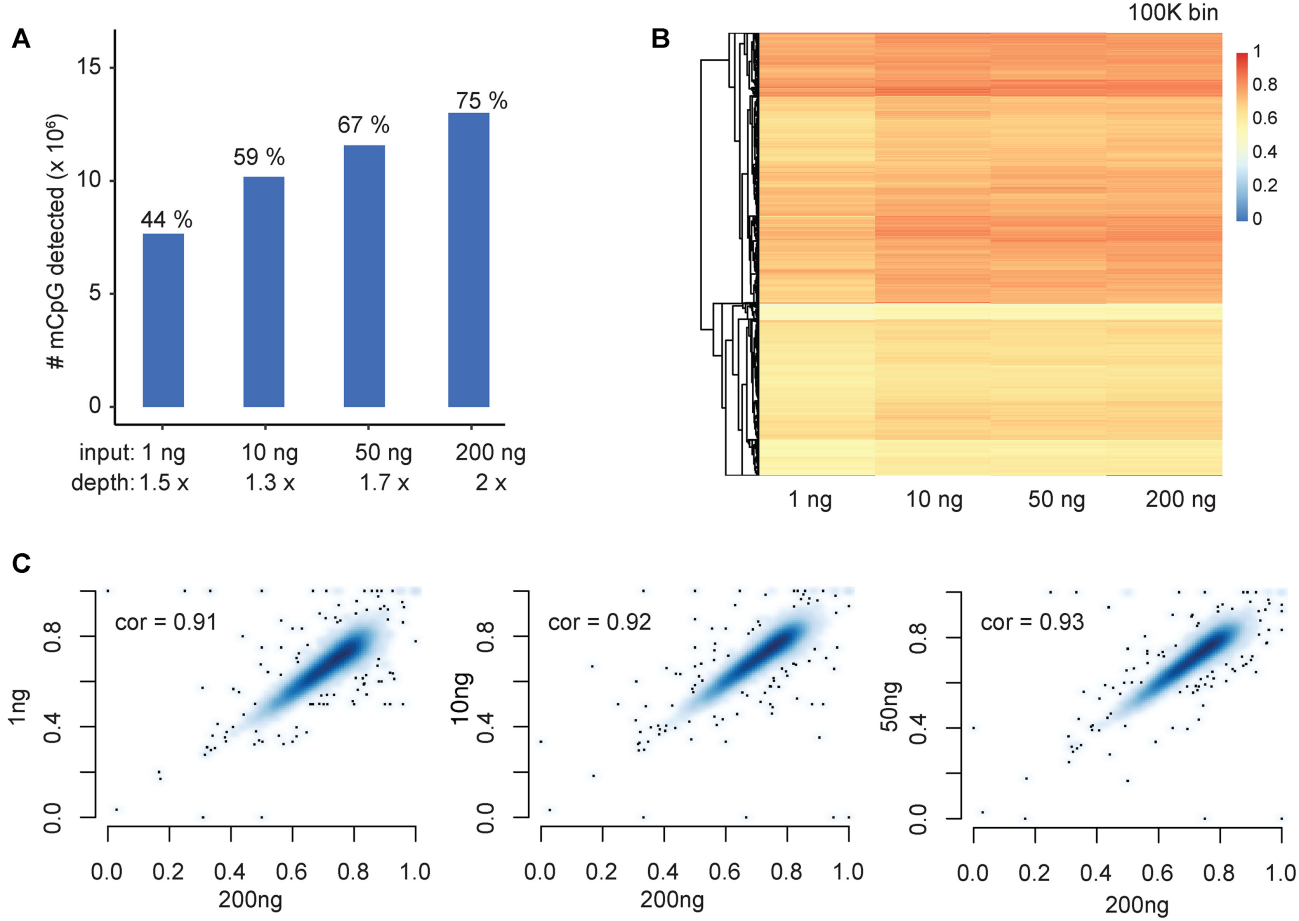
eeTAPS analysis on low-input samples. (**A**) Number of mCpGs (identified by wgTAPS) detected using eeTAPS with 1, 10, 50, 200 ng mESC gDNA input. For 200ng mESC, reads were down-sampled to 2× to match the sequence depth for low-input sample. mCpG was designated using the following criteria: Methylation level >0.1 and cleaved count ≥1 was designated as mCpG in eeTAPS. The percentages shown above the bars are the percentages of mCpG detected (mCpG detected in wgTAPS is defined as truth). (**B**) Heatmap showing eeTAPS-measured methylation distribution across the mouse genome using different input levels. Each chromosome was divided into 100 kb windows, represented by the heatmap rows. Methylation level was calculated by taking the mean methylation level across all covered CpG sites within each 100 kb window. (**C**) Density plots showing the correlation of methylation between low-input samples to the 200 ng input sample. Methylation level was calculated in 100 kb windows across the whole mouse genome as shown in (B). Pearson correlation coefficients are shown in each plot.

To evaluate the performance of eeTAPS on low-input samples, we applied it to 1, 10 and 50 ng mESC gDNA respectively. For 200 ng mESC DNA sample, sequencing reads were down-sampled to 2× to match the sequencing depth of low-input samples. We found that 44% of the mCpG sites identified by wgTAPS are also recovered using 1 ng DNA in eeTAPS. The percentage increased to 67% when 50 ng mESC DNA was used (Figure 4A). To further compare the whole genome methylation profile with these low-input samples, we binned the genome into 100 kb windows and computed the average methylation level within each bin (Figure 4B). A highly consistent methylation profile was observed among these low-input samples (with *r* = 0.91, 0.92 and 0.93 for 1, 10 and 50 ng, respectively compared to 2 × 200 ng eeTAPS, Figure 4C), thus further indicating the feasibility of eeTAPS application to low-input DNA samples.

### Effect of sequencing depth on eeTAPS

**Figure 5.**
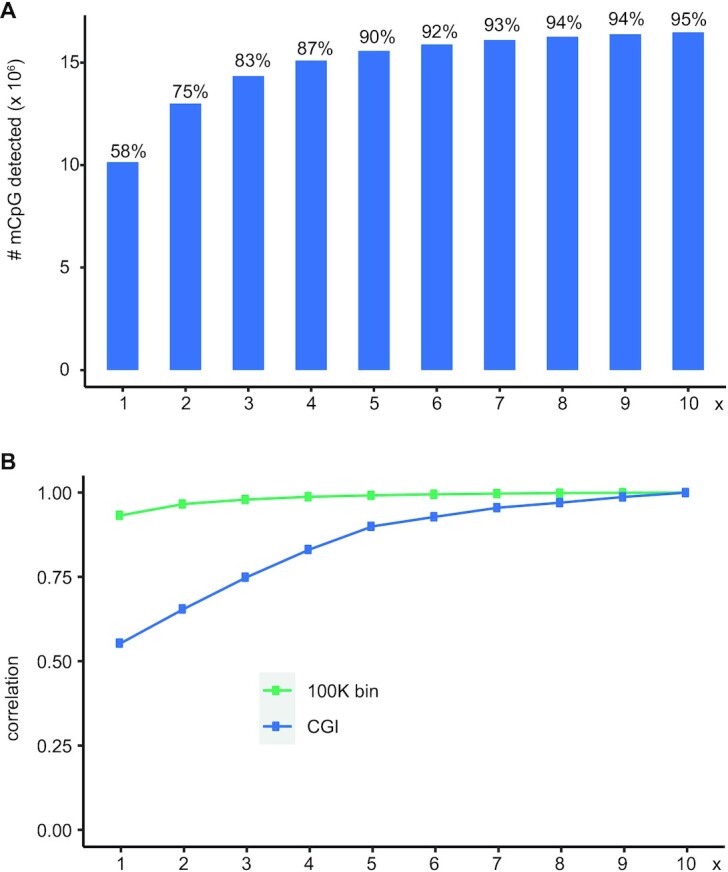
eeTAPS sequencing depth analysis. (**A**) Number of mCpGs that are detected when sampling reads from 1 to 10× sequencing depth. The percentage shown above is the percentage of mCpGs detected by eeTAPS (mCpG detected in wgTAPS is defined as truth). (**B**) The correlation of methylation in 100 kb windows across the whole mouse genome (green) and at CpG islands (CGI) (blue) when sampling reads from 1 to 10× sequencing depth.

To assess the effect of sequencing depth on the total number of mCpGs that can be detected, we down-sampled eeTAPS and evaluated the performance. As expected, the total number of detected mCpGs increased with deeper sequencing (Figure 5A). Nonetheless, with 4× (70M reads) sequencing depth, 87% mCpG sites could be successfully detected (among the 17.3M mCpG sites detected in 27× wgTAPS, 15.1M sites were also defined as mCpG in 4× eeTAPS). A similar trend was observed in terms of the methylation correlation across chromosomes and CGIs between down-sampled eeTAPS and 10× eeTAPS (Figure 5B), and Pearson correlation coefficients in CGIs reached 0.83 for 4× depth (Figure 5B). Thus, we demonstrated that eeTAPS can accurately provide a global methylation profile at a reduced sequencing cost compared to wgTAPS.

## DISCUSSION

wgTAPS could provide the most comprehensive quantitative and base-resolution whole-genome methylation. However, the steep cost of whole-genome sequencing and the large amount of data produced still limits its broad application in many projects. mCpGs constitute a minor fraction in mammalian genomes, therefore, whole genome sequencing is not the most data-efficient approach to learn about methylation status. A cost-efficient approach would be to specifically select only the regions containing mCpGs for further analysis by sequencing. Reduced-representation sequencing based on restriction enzyme digestion enrichment of CpG-rich regions and subsequent bisulfite sequencing is a cost-effective approach for methylome analysis; however, this method only covered a small proportion of CpG sites in the genome (13). TAPS is compatible with the reduced-representation approach, and we have demonstrated rrTAPS can accurately quantify methylation in a subset of the genome, especially in CGIs. Aside from the well-established biological implication of CpG methylation in gene promoters, extensive studies have also focused on intergenic DNA methylation for its potential involvement in cell fate commitment and tumorigenesis (24,25). To extend the enrichment approach to genome-wide CpG sites, we further utilized the advantage of TAPS to directly convert 5mC to DHU, which allowed DHU-cleaving endonuclease-induced cleavage at these specific modified bases. Through selective enrichment of these fragments coupled with sequencing, we demonstrated that eeTAPS enables the detection of CpG methylation on a genome-wide scale. Such a strategy is only possible because of the direct detection of methylated cytosines by TAPS. Unlike traditional antibody-based enrichment method, eeTAPS offers the possibility of direct methylation detection at single CpG resolution.

We demonstrated that eeTAPS can be used to capture genome-wide methylation signatures at single-CpG resolution in a cost-effective manner, which fills the gap between rrTAPS and wgTAPS. The eeTAPS methylation profiles across multiple different genomic features correlated well with those obtained using wgTAPS. Furthermore, with only 70 M reads, eeTAPS can detect 87% of the mCpGs detected by 27× wgTAPS. As with any enrichment-based methods, eeTAPS is only semi-quantitative at single-CpG sites. In this study, we selected fragments of 100 bp–1 kb as a proof of concept to cover a broad range of methylation density. Despite this, eeTAPS performs best in moderate methylation density region as compared to low and high methylation density regions. Nevertheless, the correlation coefficient between wgTAPS and eeTAPS was still as good as 0.55 at CpG level. Further development could use a narrower or different size selection of digested fragments, analogous to enhanced RRBS (eRRBS), to improve the performance for regions with extreme methylation density (26). Building on the mild nature of TAPS reaction, we further showed that eeTAPS is also a promising cost-effective protocol in methylation detection with low-input DNA samples.

## DATA AVAILABILITY

All sequencing data are available in GEO under GSE148197 (https://www.ncbi.nlm.nih.gov/geo/query/acc.cgi?acc=GSE148197). The code used to process eeTAPS data can be downloaded from https://gitlab.com/jfeicheng/userenrich.

## Supplementary Material

gkab291_Supplemental_Files

## Notes

Present address: Yibin Liu, Exact Sciences Innovation, Innovation Building, Oxford OX3 7FZ, UK.
