# Supplementary material for: Endonuclease enrichment TAPS for cost-effective genome-wide base-resolution DNA methylation detection
Source: Nucleic Acids Res. 2021 Apr 27;49(13):e76. doi: 10.1093/nar/gkab291 (PMC8287915; doi:10.1093/nar/gkab291)
Supplement: gkab291_Supplemental_Files [file gkab291_supplemental_files.zip › eeTAPS supplementary figures.pdf]

Supplementary information for

Endonuclease enrichment TAPS for cost-effective genome-wide base-resolution DNA methylation detection

Jingfei Cheng[1,2]*, Paulina Siejka-Zielińska[1,2]*, Yibin Liu[1,2,3], Anandhakumar Chandran[1], Skirmantas Kriaucionis[1], Chun-Xiao Song[1,2]†

[1]Ludwig Institute for Cancer Research, Nuffield Department of Medicine, University of Oxford, Oxford OX3 7FZ, UK
[2]Target Discovery Institute, Nuffield Department of Medicine, University of Oxford, Oxford OX3 7FZ, UK
[3]Present address: Exact Sciences Innovation, Innovation Building, Oxford OX3 7FZ, UK.

*These authors contributed equally to this work.
†Corresponding author. E-mail: chunxiao.song@ludwig.ox.ac.uk (C.-X.S.).

Supplementary figures

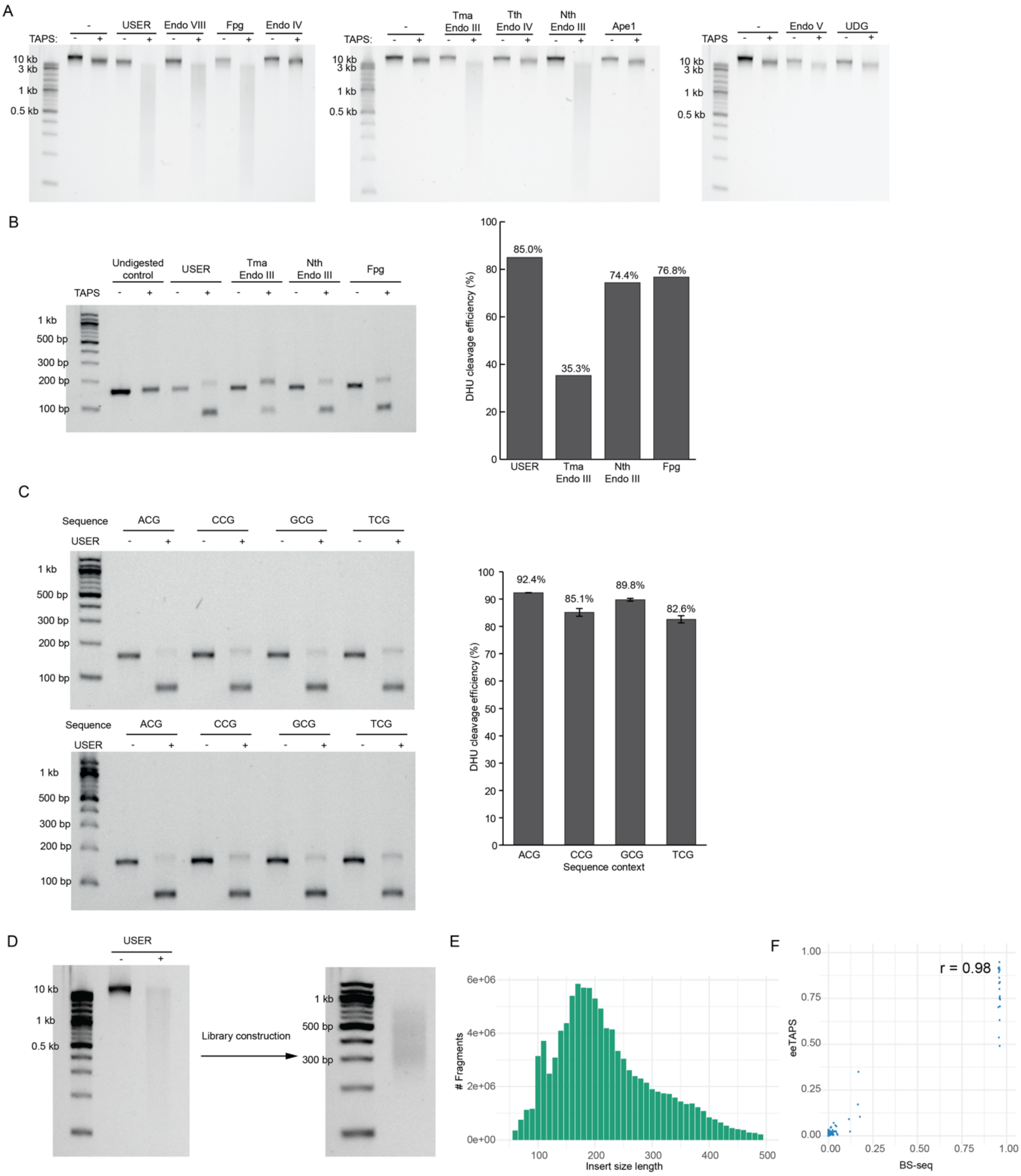

Supplementary Figure 1.

A. Results of mESC gDNA digestion by different endonucleases before and after TAPS conversion. TAPS conversion introduces DHU in place of methylated cytosine. Endo VIII - endonuclease VIII, Endo IV - endonuclease IV, Tma Endo III - endonuclease III,

Tth Endo IV - endonuclease IV; Nth Endo III - Nth endonuclease III; Endo V - Endonuclease V.

B. Quantification of DHU cleavage efficiency by selected endonucleases based on digestion of unconverted and TAPS-converted model DNA containing single mCpG. Cleavage efficiency was estimated based on image bands quantification. Digestion of unconverted DNA substrate was not observed for any enzyme. USER enzyme exhibited the best DHU cleavage efficiency of 85.0% in TAPS-converted model DNA.

C. Quantification of DHU cleavage efficiency by USER in different sequence contexts (ACG, CCG, GCG and TCG). Cleavage efficiency was estimated based on image bands quantification. Bar plot shows average DHU cleavage efficiency by USER enzyme depending on sequence contexts. Values above bars show average cDHU cleavage efficiency based on two independent experiments. Error bars represent standard error.

D. Representative image of TAPS-treated mESC gDNA before USER digestion, after USER digestion, and after library construction.

E. Insert size distribution of eeTAPS library. Fragments with insert size 50-500 were plotted.

F. Scatter plot showing the methylation level in all CpGs measured by both BS-seq and eeTAPS in 4 kb model DNA.

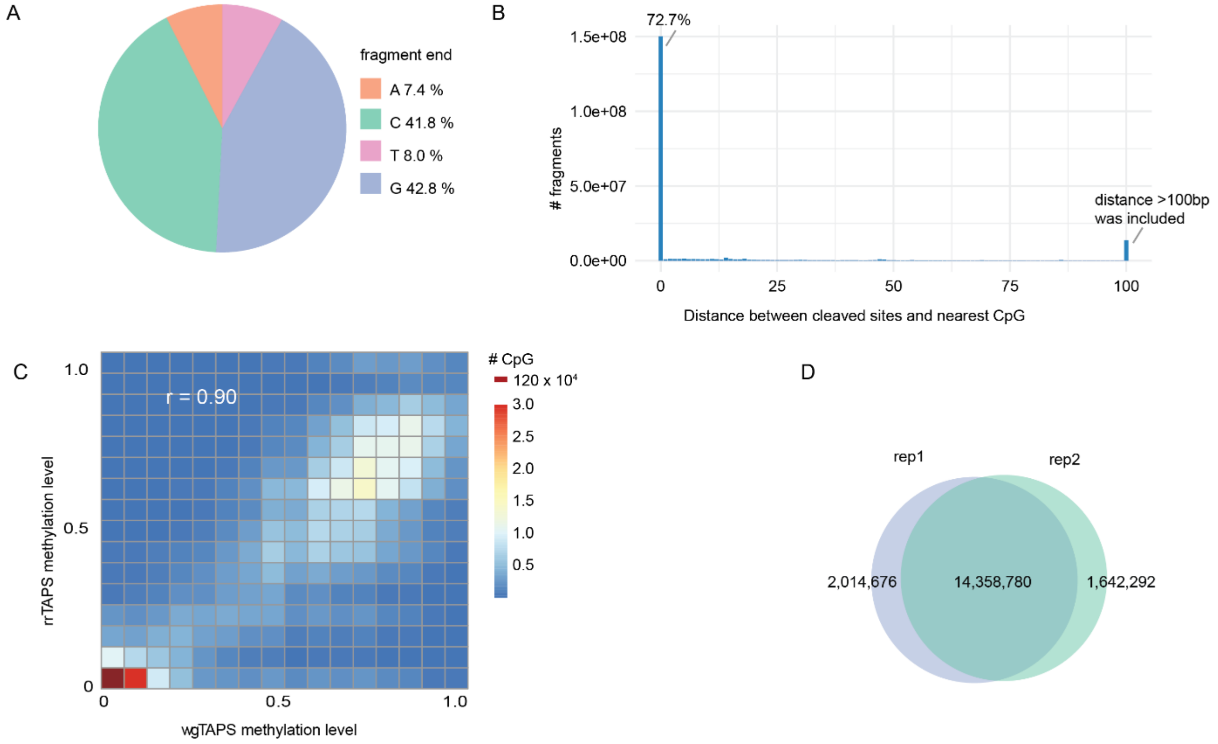

Supplementary Figure 2.

A. Nucleotide frequency at the ends of the sequenced fragments.

B. Bar plot showing the distribution of distance between cleaved sites and its nearest CpG.

C. Heatmap showing the correlation of methylation level determined by wgTAPS and rrTAPS at single CpG-resolution. The methylation level was divided into 16 group for both wgTAPS and rrTAPS. The colour shows the number of CpGs in specific intervals. Only sites with wgTAPS coverage >= 5 and rrTAPS coverage >= 5 were taken into consideration. The Pearson correlation coefficient is 0.90.

D. Overlap of mCpG sites detected in replicates of eeTAPS, replicates were sub-sampled to the same depth to detect mCpG.

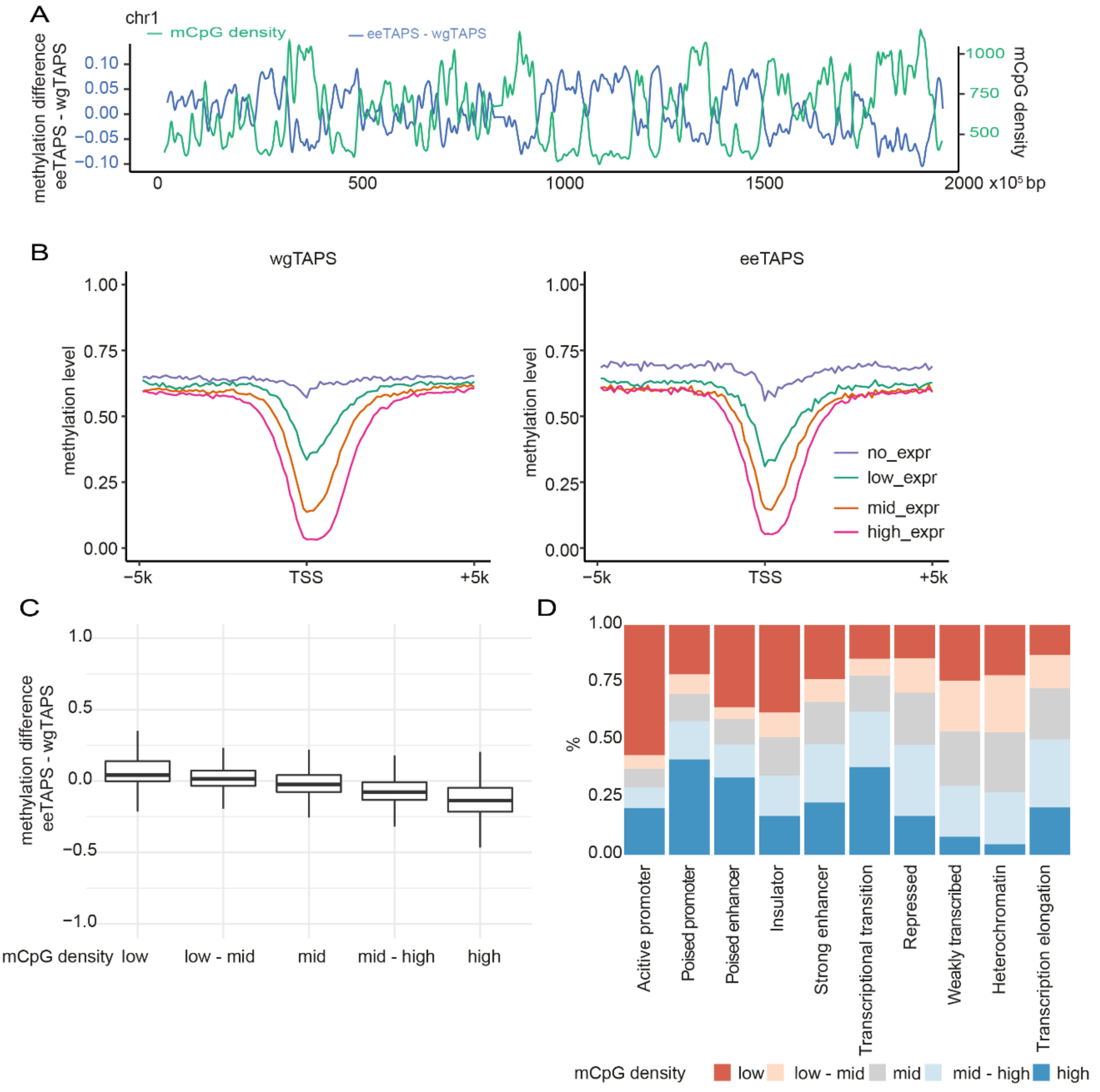

Supplementary Figure 3.

A. Methylation difference between eeTAPS and wgTAPS and mCpG density distribution across chromosome 1 of the mESC. CpG sites with methylation level >=0.1 were defined as mCpG, and the number of mCpG in 100 kb windows were used as mCpG density. A moving average value was calculated using the movAvg2 function in R with bw =10.

B. Average methylation distribution around Transcription start sites (TSS) in wgTAPS and eeTAPS. Genes were categorized by their expression level according to GSE72855 dataset.

C. Methylation difference between eeTAPS and wgTAPS and mCpG density distribution across all chromatin features. mCpG density was defined as mCpG / (region length). Regions were categorized into quintiles by the mCpG density.

D. Barplot showing the percentage of mCpG density group across different chromatin features.
